# Left-Sided Amyand’s Hernia Managed Without Appendectomy: A Case Report of a Rare Diagnosis

**DOI:** 10.7759/cureus.82559

**Published:** 2025-04-19

**Authors:** Leonor Murça da Silva Balo, Rafael Antônio Vicente Lacerda, Damião Maquina Mariti, Joaquim Lima Quibuco, Yunior Collazo Velazco

**Affiliations:** 1 Department of Surgery, Hospital Militar Principal/Instituto Superior, Luanda, AGO; 2 Department of Internal Medicine, Medical School of Juazeiro do Norte, Juazeiro do Norte, BRA; 3 Department of Surgery, Hospital Clínico Quirúrgico Docente “Dr. León Cuervo Rubio”, Pinar del Río, CUB

**Keywords:** giant amyand's hernia, hernia repair, incarcerated inguinal hernia, position of appendix, rare form inguinal hernia

## Abstract

Hernias are defined as protrusions of an organ or its fascia through the wall of a cavity. Amyand's hernia, in turn, is a rare subtype in which the vermiform appendix is part of the hernial content. We report the case of a 45-year-old Angolan patient with a large left-sided Amyand’s hernia, which appeared in childhood at the age of 5, with progressive enlargement and an inability to manually reduce the contents for the past seven years. He was referred from the Hospital Militar Principal/Instituto Superior in Luanda, Angola. Large inguinoscrotal hernias remain frequent in African countries, often associated with limited access to, or late-seeking of, healthcare services. Delayed management of these patients is linked to an increased risk of preoperative complications, such as incarceration, strangulation, appendicitis, and perforation-induced peritonitis, as well as postoperative complications, including abdominal compartment syndrome. Therefore, early diagnosis and individualized patient management - considering age and comorbidities, especially in the early stages - are of utmost importance for effective and less traumatic correction.

## Introduction

Abdominal hernias are among the most common surgical conditions, characterized by the protrusion of an organ or tissue through an abnormal opening in the abdominal wall [1]. They may arise due to congenital or acquired weakening of the musculature, often associated with factors such as excessive physical exertion, obesity, pregnancy, or previous surgeries. Hernias vary in location and clinical presentation, with the most common types being inguinal, umbilical, incisional, and epigastric hernias [1]. While often asymptomatic in their early stages, hernias can progress to severe complications such as incarceration or strangulation, requiring urgent medical intervention [1,2].

Among these, inguinal hernias are the most prevalent type of abdominal hernia, accounting for a significant proportion of surgically treated conditions worldwide. They occur when intra-abdominal contents such as intestinal loops or adipose tissue protrude through the inguinal canal due to a weakening or defect in the abdominal wall [3].

The presence of the vermiform appendix within an inguinal hernia was first described in 1735 by Claudius Amyand. Since then, "Amyand’s hernia" has been recognized in the literature as a rare condition, with an estimated prevalence of approximately 1% [4]. Most cases of Amyand’s hernia are diagnosed incidentally during surgery [5,6]. The right inguinal region is the most common site, owing to the natural anatomical position of the appendix, which is connected to the cecum in the right iliac fossa [5,7]. Its occurrence on the left side is even rarer, with only five cases reported in the past 25 years [3,4]. Inflammation of the appendix within the inguinal hernia sac is also uncommon, representing only 0.1% (0.07%-0.13%) of all Amyand’s hernia cases and 2% of appendectomies in the neonatal and pediatric populations [7,8].

This study was conducted in accordance with the CARE (CAse REport) guidelines, highlighting the importance of a tailored approach in the management of complex cases. We report a particularly rare case of a patient with a large left-sided Amyand’s hernia, which developed over approximately 40 years and became incarcerated seven years ago, and was surgically repaired without appendectomy. A discussion follows, comparing the case with the existing literature through a detailed analysis of the hernia characteristics (size, laterality, duration, and incarceration), patient factors (age and absence of comorbidities), and surgical technique (from preoperative assessment considerations to intraoperative decision-making).

## Case presentation

Preoperative period

This case involves a 45-year-old Black male patient with a progressive increase in volume in the left inguinoscrotal region since childhood, approximately 40 years ago. Initially reducible and painless, the swelling gradually enlarged, extended toward the anterior genicular region, and became irreducible seven years ago. The patient experienced occasional colic pain, a sensation of heaviness in the scrotal region, and limitations in ambulation. Due to significant impairment in daily activities and social stigmatization, he sought medical assistance at the Hospital Militar Principal/Instituto Superior in Luanda, Angola.

Upon admission, the patient was conscious, alert, and cooperative, though he presented with poor hygiene conditions. He had moist, normochromic mucosae; anicteric sclerae; and was afebrile, eupneic, and hemodynamically stable. He did not report pain or bowel or urinary changes. On physical examination, his extremities were normal, without edema. The abdomen was flat, moved with respiration, and had present, normal bowel sounds. It was soft and non-tender on superficial and deep palpation, with no palpable masses or organomegaly. The cardiovascular and pulmonary examinations were normal.

**Figure 1:**
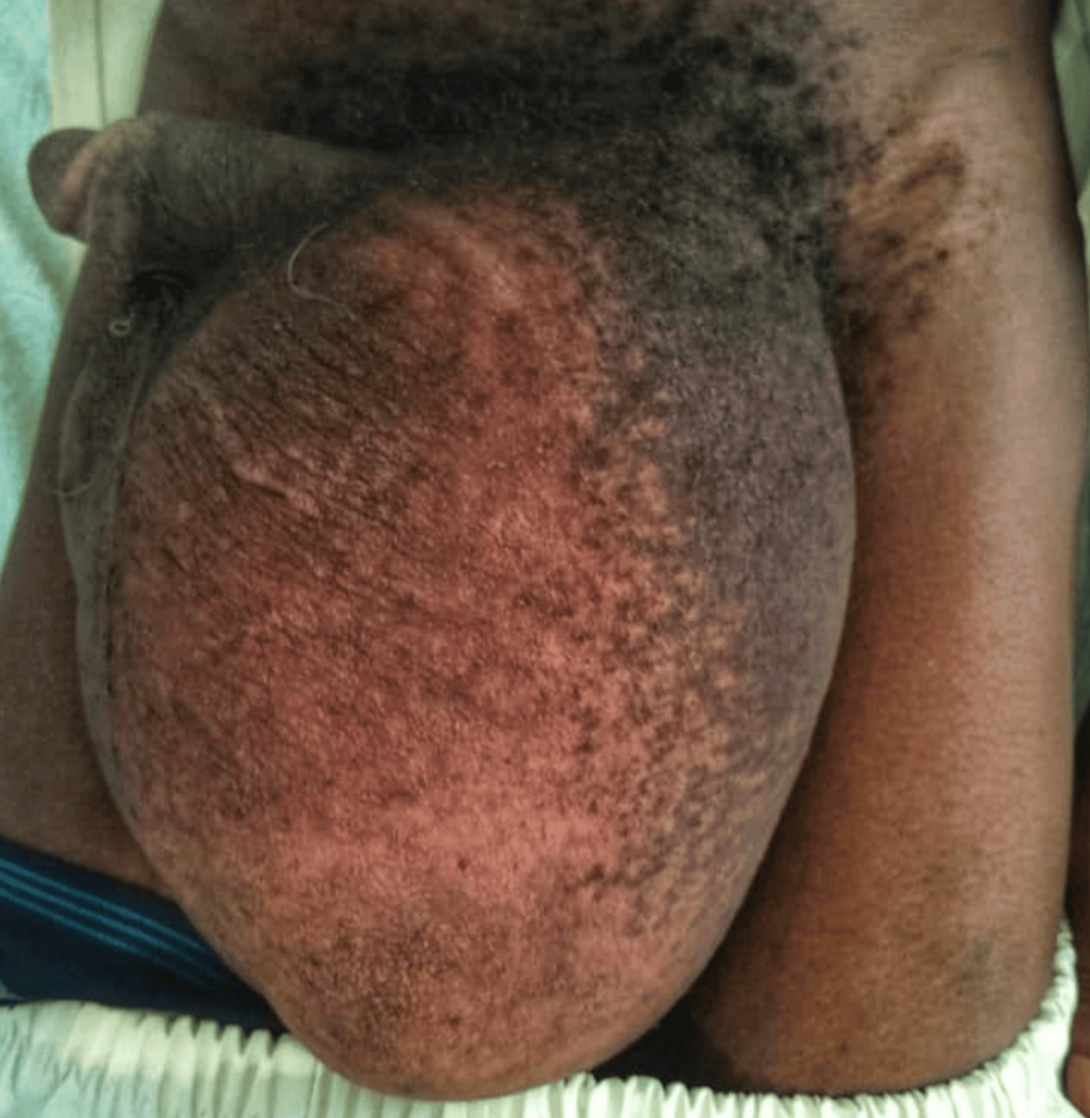
Preoperative period A large, non-reducible left inguinoscrotal hernia, extending approximately 5 cm to the anterior knee region.

The inguinal region exhibited marked asymmetry due to the enlarged left inguinoscrotal mass, extending from the left inguinal region to approximately 5 cm above the anterior knee. The area presented skin hyperpigmentation, with excoriations on the inner thigh, but no signs of infection. The scrotal sac showed visible peristalsis, with normal bowel sounds on auscultation, a negative transillumination sign, and a slightly buried penis. On palpation, the mass was firm but elastic, irreducible to manual taxis, non-tender, and exhibited a difficult Landivar maneuver due to the occupied external inguinal ring (Figure 1).

The ultrasonography revealed a large left inguinoscrotal hernia containing visible peristaltic intestinal loops. The testes were in a normal anatomical position, with normal dimensions and regular contours, though the left testis exhibited decreased echogenicity, minimal hydrocele, and adjacent subcutaneous thickening, suggesting an inflammatory process. The laboratory tests revealed only mild normocytic and normochromic anemia. There was no leukocytosis or abnormalities in platelet count. Transaminases, renal function, and coagulation profile were within normal limits, and there was no evidence of infection with human immunodeficiency virus, hepatitis B or C viruses, or syphilis.

Intraoperative period

**Figure 2:**
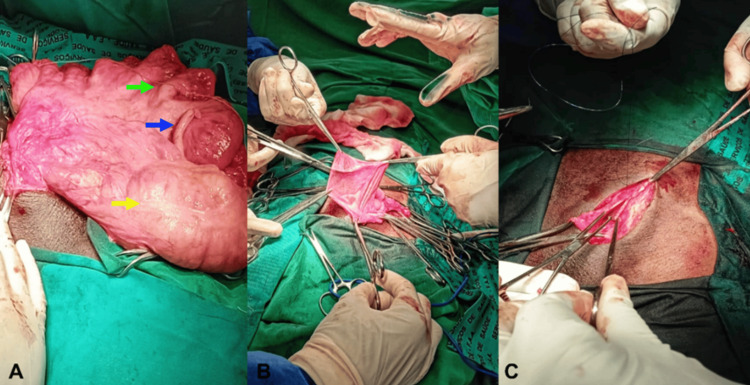
Intraoperative findings and technique A) Opened scrotal sac containing the small intestine (green arrow), right colon (yellow arrow), and cecal appendix (blue arrow); B) Ombredanne’s maneuver; C) Lichtenstein technique.

Under general anesthesia, an aseptic surgical approach was performed with an extended left oblique inguinotomy approximately 7 cm in length. Dissection proceeded through anatomical planes until the external oblique aponeurosis was opened. Due to the inability to mobilize the spermatic cord, the scrotal sac was opened, revealing its contents: small intestine, right colon, and the cecal appendix (Figure 2A). Reduction of the contents into the abdominal cavity required enlargement of the deep inguinal ring. To manage the scrotal sac, the Ombredanne maneuver was performed (Figure 2B). The deep inguinal ring was reconstructed using non-absorbable sutures, and posterior wall repair was achieved with the polypropylene mesh using the Lichtenstein technique (Figure 2C), followed by hemostasis and layered closure of the surgical wound.

Postoperative period

**Figure 3:**
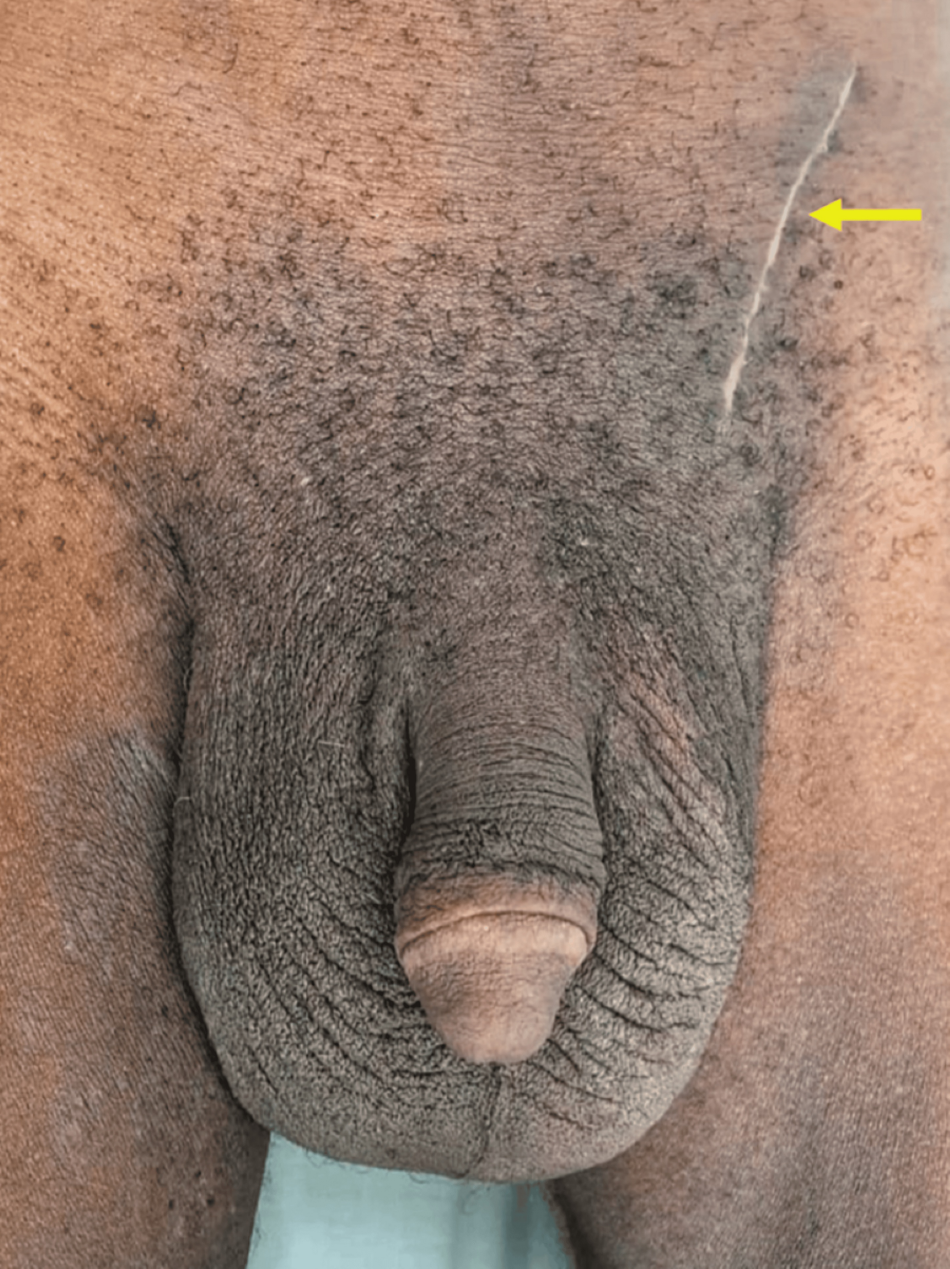
Postoperative period The yellow arrow depicts the left hernioplasty scar.

The patient had a satisfactory postoperative course with minimal inguinal pain and was discharged on the fourth postoperative day with prescriptions for analgesics, anti-inflammatory medication, relative rest, and supportive scrotal garments. He continued outpatient follow-up at our institution and, on the 58th postoperative day, showed no surgical complications, with excellent functional (ambulation) and aesthetic recovery (Figure 3).

## Discussion

Amyand's hernia is defined as the presence of the vermiform appendix within the inguinal hernia sac, with a true prevalence of approximately 1%. It is more commonly found on the right side in patients with long-standing hernias [4,5,7,9]. The right-sided predominance is explained by the natural anatomical location of the appendix and the higher prevalence of right inguinal hernias. Consequently, left-sided Amyand’s hernia is even rarer and is often associated with situs inversus, mobile cecum (as in the reported case), intestinal malrotation, or an unusually long appendix [3,8,10]. This type of hernia is three times more common in children and males due to the persistence of the processus vaginalis [6]. In line with the literature, the patient in this case was male and had the hernia since childhood. The left inguinofemoral hernia contained the vermiform appendix, ileum, and right colon, likely due to the large hernia volume resulting from extensive posterior wall destruction and deep inguinal ring dilation over 40 years.

Most cases of Amyand’s hernia are diagnosed incidentally during surgery [5,6]. However, preoperative imaging is crucial in defining the best treatment strategy [1]. Consistent with the literature, the initial ultrasound in this case reported only intestinal loops, without mention of the colon or vermiform appendix, highlighting the diagnostic limitations of preoperative imaging [4,6,11]. The patient’s clinical presentation included chronic colic abdominal pain relieved by defecation, a sensation of scrotal heaviness, and difficulty in ambulation. There were no features of acute inflammatory or obstructive abdomen, which are the most common presentations of Amyand’s hernia [9].

Globally, patients with such large inguinoscrotal hernias are rare. However, in Africa, this condition remains prevalent due to a healthcare system still in development and the population’s low socioeconomic status [12]. Surgical approaches to long-standing, large hernias carry higher risks of complications. Age over 70, fever, signs of obstructive acute abdomen, peritonitis, and leukocytosis are frequently associated risk factors [13]. Contrary to the literature, the patient in this case presented none of these factors, allowing surgical intervention without appendectomy.

Repair of large hernias with loss of domain requires careful analysis of patient comorbidities, potential risks, and possible postoperative complications, such as abdominal compartment syndrome. To reduce this risk, the literature describes preoperative techniques, such as progressive pneumoperitoneum and botulinum toxin injections, to increase abdominal cavity capacity before hernia repair, allowing better physiological adaptation postoperatively [14,15]. This patient had an irreducible hernia for seven years, extending to approximately 5 cm below the knees, but without comorbidities. Therefore, these techniques were not applied, and direct hernia content reduction was performed with continuous monitoring for early detection of abdominal compartment syndrome.

Additionally, according to the Losanoff and Basson classification (Table 1), appendectomy in type 1 Amyand’s hernia is optional [16]. Specifically, for left-sided hernias, some studies suggest appendix removal regardless of its condition, as future appendicitis would present atypically and could cause diagnostic confusion [8]. However, in this case, the risk of secondary infection due to possible stump dehiscence during hernia content reduction and the associated increased likelihood of abdominal compartment syndrome led to the decision not to perform an appendectomy. Instead, hernioplasty was performed using the Lichtenstein technique.

**Table 1:** Losanoff and Basson Classification Source: [16]

Types of hernia	1	2	3	4
Characteristic of the content	Normal appendix	Acute appendicitis in the hernia sac	Acute appendicitis with peritonitis	Appendicitis + another abdominal pathology
Surgical management	Reduction into the cavity without appendectomy AND hernioplasty OR appendectomy AND hernioplasty using the Lichtenstein technique	Appendectomy via inguinotomy AND (open herniorrhaphy using Bassini or Shouldice technique)	Appendectomy via exploratory laparotomy AND open herniorrhaphy	Appendectomy via exploratory laparotomy AND treatment of the underlying cause AND open herniorrhaphy

There is significant controversy regarding the surgical approach to Amyand’s hernia, emphasizing the need to assess the patient’s profile, particularly regarding comorbidities and hernia characteristics. Laparoscopic repair is widely used in children, with favorable clinical outcomes and shorter hospital stays [5,6]. Some authors have reported similar and satisfactory results regardless of the approach used for hernioplasty, even in type 2 Amyand’s hernias, challenging the fixed recommendation by Losanoff and Basson for open repair in such cases [8,17-20]. In this case, due to the hernia’s large volume and prolonged incarceration, we opted to follow the Losanoff and Basson classification recommendations and performed an open Lichtenstein hernioplasty.

## Conclusions

Left-sided Amyand’s hernia is a rare condition that demands high clinical suspicion and a tailored management approach. This case emphasizes the feasibility of appendix preservation in the absence of inflammation, even in large hernias, highlighting the importance of individualized surgical decisions. The Ombredanne maneuver and Lichtenstein technique were pivotal for effective management of the hernial sac and posterior wall reinforcement. Additionally, hernia volume should be considered when selecting the surgical approach, as it may influence the ease of repair. Indeed, this case exemplifies the core principle of family and community medicine: "We treat patients, not just diseases." A comprehensive assessment of the patient should always serve as the guiding principle for defining a tailored and, therefore, safer approach. Further research is warranted to refine treatment algorithms and establish evidence-based guidelines for managing complex cases of Amyand’s hernia.
